# Three Recombinant Engineered Antibodies against Recombinant Tags with High Affinity and Specificity

**DOI:** 10.1371/journal.pone.0150125

**Published:** 2016-03-04

**Authors:** Hongyu Zhao, Ao Shen, Yang K. Xiang, David P. Corey

**Affiliations:** 1 Department of Neurobiology and Howard Hughes Medical Institute, Harvard Medical School, Boston, Massachusetts, United States of America; 2 Department of Pharmacology, School of Medicine, University of California, Davis, California, United States of America; King's College London, UNITED KINGDOM

## Abstract

We describe three recombinant engineered antibodies against three recombinant epitope tags, constructed with divalent binding arms to recognize divalent epitopes and so achieve high affinity and specificity. In two versions, an epitope is inserted in tandem into a protein of interest, and a homodimeric antibody is constructed by fusing a high-affinity epitope-binding domain to a human or mouse Fc domain. In a third, a heterodimeric antibody is constructed by fusing two different epitope-binding domains which target two different binding sites in GFP, to polarized Fc fragments. These antibody/epitope pairs have affinities in the low picomolar range and are useful tools for many antibody-based applications.

## Introduction

Antibodies with good affinity and specificity are critical tools for biomedical research. As a recent policy proposal points out, however, polyclonal antibodies are often neither very specific nor reproducible, and monoclonal antibodies may also have poorly-defined specificity [1]. Recombinant antibodies derived from a known coding sequence are much more reproducible and they can be engineered to have higher affinity, specificity and stability. However some methods of improving affinity, involving multiple rounds of mutation and selection, can be very time-consuming.

**Fig 1:**
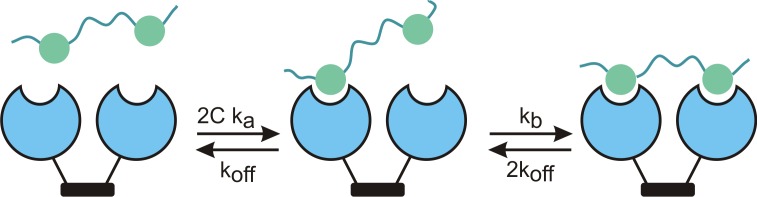
Schematic of binding and unbinding for a divalent antibody and tandem epitope. A tandem epitope (green) with variable linker length is bound in two steps by a divalent antibody (blue). If the proximity-dependent rebinding rate (k_b_) is much larger than the monovalent unbinding rate (k_off_) then avidity is greatly increased.

Bi-specific antibodies against a single protein (or protein complex) have been used to increase effective affinity (avidity) and specificity through tandem binding [2] [3] [4]. Antibodies are normally divalent, and tandem epitopes can exploit this to increase the avidity (Fig 1).

The rate for binding one epitope of a tandem pair, Ck_a_ (where k_a_ is the concentration-dependent on rate), is only doubled by the presence of two epitopes. However the rate from the singly-bound to the doubly-bound state, k_b_, is hugely increased because the effective concentrations of the second binding domain and epitope are determined by their close proximity rather than by solution concentrations. Although there are detailed treatments of effective molarity that incorporate a random-coil polymeric linker between epitopes [5], a simple calculation is based on the volume accessible to the second epitope as determined by linker length. For instance a particle constrained within a 20-nm radius has an effective molarity of about 50 μM. At the same time, the unbinding rate is nearly unchanged, with a rate from doubly to singly bound, 2k_off_, that is just twice the monomeric off rate. Thus if one epitope unbinds, it is much more likely to rebind before the second epitope unbinds. The effective dissociation constant can be much lower, often in the picomolar range. This effect is more pronounced if the effective radius is smaller, and less pronounced it the monomeric dissociation constant is higher. Finally, it would not occur for off-target binding, where the inappropriate epitope is usually not in tandem. Divalent binding thus increases both avidity and specificity.

We report the generation of three recombinant antibodies with high affinity for an engineered epitope, using fusion proteins that include a small epitope-binding domain (EBD) and that dimerize through standard antibody Fc domains. In one strategy two identical binding domains independently bind to two identical epitopes placed in tandem with a short peptide linker. These tandem epitopes may be inserted into a protein of interest for localization or immunoprecipitation experiments. In a second strategy, two different EBDs bind to two different regions of a single, untagged target protein, but are linked as a heterodimer by polarized Fc domains.

Previously reported bi-specific antibodies are generally against specific antigens rather than engineered epitope tags [2] [3] [4]. These often use a flexible linker or a leucine zipper between the two EBDs. The antibodies reported here use Fc domains from mouse or human to form natural, covalent dimers. The Fc portion also helps to improve protein solubility and yield during expression, and can be easily purified using protein A/G. It also serves as a handle to which secondary antibodies bind and to which other secondary components (fluorescent dyes, gold particles, etc.) may be conjugated.

Finally, we show that avidity depends on the length of the linker between the tandem epitopes, reflecting steric constraints in EBD binding that can reduce the theoretical improvement in avidity.

## Materials and Methods

### Theory

The population of the unbound (P_0_), singly-bound (P_1_) and doubly-bound (P_2_) states of the antibody can be calculated by the general form of rates for a three-state system:

$$P_{1}=1/\left(1+\frac{k_{12}}{k_{21}}+\frac{k_{10}}{k_{01}}\right)$$ (1)

$$P_{0}=\frac{k_{10}}{k_{01}}P_{1}$$ (2)

$$P_{2}=\frac{k_{12}}{k_{21}}P_{1}$$ (3)

where *k*_01_ (= 2C*k*_a_ in Fig 1) and *k*_12_ (= *k*_b_) are concentration-dependent binding rates with *k*_01_ depending on solution concentration and *k*_12_ depending on effective concentration calculated from accessible volume. For these reagents, the volume has a radius of ~30 nm. *k*_21_ and *k*_10_ are concentration-independent off rates and *k*_12_ is twice *k*_10_ to reflect the possibility of either half of a tandem epitope unbinding. Fraction bound is (P_1_ + P_2_)/ (P_0_ + P_1_ + P_2_).

### Construction of tandem epitope-tagged proteins

*Tandem GCN4*. Sequences encoding the 12-aa GCN peptide in tandem with a 10-aa or 19-aa linker, together with the surrounding PCDH15 coding sequence, were synthesized and inserted into protocadherin-15 (PCDH15) cDNA between EcoNI and EcoRV sites, resulting in a protein product with an insertion between EC domain 10 and EC domain 11.

*THAP*. Sequences encoding the high affinity peptide (HAP), or HAP in tandem with a 10-aa or 14-aa linker, together with the surrounding PCDH15 coding sequence, were synthesized and inserted into protocadherin-15 (PCDH15) cDNA between EcoNI and EcoRV sites, resulting in a protein product with an insertion between EC domains 10 and 11.

### Construction of Fc-fusion antibodies

*C11L34-Fc*. A codon-optimized cDNA sequence coding for the C11L34 scFv was synthesized and sub-cloned (using Gibson Assembly) into the EcoRI/BglII site of the pINFUSE vector (Invivogen) containing the mouse IgG2b Fc, to create a homodimeric C11L34-Fc fusion.

*anti-THAP*. Codon-optimized cDNA sequences coding for α bungarotoxin (PDB: 1KL8_A) were synthesized and sub-cloned into the EcoRI/BglII site of the pINFUSE vector containing the human IgG2b Fc.

*GBP1*, *GBP6-Fc*. pINFUSE vectors containing the human IgG1 Fc were mutated to generate heterodimer-forming constructs, pINFUSE_hIgG1_DD and pINFUSE_hIgG1_KK [6].

Codon optimized cDNA sequences coding for GBP1 or GBP6 were synthesized and sub-cloned into the EcoRI/BglII site containing the human IgG1 Fc.

Production and purification were performed by the University of North Carolina at Chapel Hill Protein Expression and Purification Core Facility. Briefly, HEK293f cells (Invitrogen) were transiently transfected (using PEI) with these pINFUSE plasmids. The cells were grown in Freestyle 293 media (Gibco) at 37°C/8%CO_2_ in suspension culture for 7–10 days. The supernatant was harvested, and secreted Fc-fusion proteins were purified using a 1 ml rProtein A HiTrap column (GE Healthcare) on an AKTA ExPress FPLC (GE Healthcare). Eluted fractions from the affinity purification were pooled and the integrity of the proteins was verified by reducing and non-reducing SDS-PAGE analysis (Novex-Invitrogen).

### Western blot

From HEK293 cells transfected (Effectene, Qiagen) with PCDH15, PCDH15-HAP, PCDH15-THAP, PCDH15-GCN, PCDH15-2XGCN_L10, and PCDH15-2XGCN_L19 cDNAs, total cell lysates were prepared and insoluble matter pelleted. Equal amounts of the supernatant were separated by 3–7% Tris-acetate SDS-PAGE (Novex-Invitrogen). Separated proteins were transferred to PVDF membranes (Immobilon FL, Millipore). For subsequent steps, we followed the protocol from Li-COR Biosciences, with overnight primary antibody incubation at 4°C (Rabbit anti-PCDH15, 0.5 μg/ml; anti-THAP, 0.01 μg/ml) and secondary antibody incubation at room temperature for 30 min. The rabbit anti-PCDH15 antibody was generated using the same synthesized peptide used by Kazmierczak et al.[7] to produce the PB811 antibody.

### Fluorescence imaging and analysis

RPMI-2650 cells (ATCC #CCL-30) were transfected (Effectene, Qiagen) with the tagged PCDH15 construct. 24–48 hours after transfection, live cells were incubated for 30 min at 4°C with anti-tag antibodies at a range of concentrations. We used C11L34-Fc, 10 pM– 30 nM; anti-THAP, 6 pM– 12 nM; BTX-biotin (Biotium), 1–150 nM. Cells were washed with PBS, fixed with 4% formaldehyde, washed with PBS, then blocked with PBS containing 10% normal goat serum (NGS) and 0.2% saponin. In some experiments they were then incubated with anti-PCDH15 antibody (using a single, fixed concentration: rabbit anti-PCDH15, 5 μg/ml). Alternatively, the transfected cells were fixed, blocked, and then incubated with both the anti-tag antibodies (using a range of concentrations) and anti-PCDH15 antibody (using a single, fixed concentration: rabbit anti-PCDH15, 5 μg/ml). CHO cells were transfected with TMC1_EGFP (a construct that expresses a fusion protein of EGFP and the transmembrane protein TMC1), fixed and blocked as above, and labeled with rabbit anti-GFP (A-11122, Life Technologies, at 5ug/ml) and/or GBP1_6 (100 pM). Cells were then incubated with secondary reagents (Jackson ImmunoResearch, goat anti-rabbit/Alexa 647, goat anti-human Fc/Cy3, and streptavidin/Alexa 488), then washed, mounted, and imaged with an Olympus FV1000 confocal microscope equipped with a 60x, 1.42 NA oil objective.

For apparent affinity calculation, 5–10 thin processes from 10–20 cells were selected for each concentration using an algorithm developed by Dr. Tiao Xie at the HMS Image and Data Analysis Core. To avoid signal saturation, only thin processes of the cells were used for quantitative analysis. The ratio of the fluorescence signal generated by the anti-tag antibody and the anti-PCDH15 antibody was calculated and averaged for each concentration of anti-tag antibody, as in McCann et al [8], Pollard [9], and Hulme & Trevethick [10]. The ratios were plotted against concentration of anti-tag antibody using GraphPad Prism software and fitted by a single exponential binding curve. From the binding curve, K_d_ and n were calculated using the following equation:

$$Y_{\min}+(Y_{\max}-Y_{\min})(\frac{L_{t}{}^{n}}{L_{t}{}^{n}+K_{d}})$$ (4)

where Y_min_ is the ratio of fluorescent signals (anti-tag/anti-PCDH15) in the absence of anti-tag antibody, Y_max_ is the ratio at saturating concentrations of anti-tag antibody, L_t_ is the total concentration of anti-tag antibodies titrated and n is the Hill coefficient. The calculation was done by Prism software.

### Surface plasmon resonance analysis

SPR experiments were carried out at the University of Maryland School of Medicine Biacore Facility, using a Biacore 3000 instrument (GE Healthcare). Flow cells of CM5 sensor chips (GE Healthcare) were coupled with goat anti-human IgG to 5,000 to 22,000 RU using amine coupling chemistry, as described by the manufacturer. The coupling was performed by injecting 30 μg/ml of goat anti-human IgG (Jackson ImmunoResearch) in 10 mM sodium acetate pH 5.0 (GE Healthcare). All anti-tag antibodies with human Fc were captured by goat anti-human IgG to a CM5 chip. HBS-EP buffer (10 mM HEPES, 150 mM NaCl, 3 mM EDTA, 0.05% surfactant P20; GE Healthcare) was used as running buffer and dilution buffer. Aliquots of 100 μl containing concentrations of each of the peptides (HAP, THAP_10, THAP_14) or recombinant EGFP (Kerafast, FR0008), ranging from 0 to 10 nM, were injected over immobilized anti-THAP or GBPs (with human Fc) at a flow rate of 50 μl/min at 25°C. Regeneration of the surfaces was done using injections of 10 mM glycine, pH 1.75, followed by HBS-EP at pH 7.4. In all experiments, data were zero adjusted and the reference cell subtracted. Data were evaluated using BIAevaluation 4.1 software (GE Healthcare).

### Single-molecule pull-down

The monomeric YFP (mYFP) construct was described previously [11]. The THAP tag was created by annealing two complementary oligos and inserted into the pCDNA3.1+ vector (Invitrogen). The mYFP-His6 cassette from the original mYFP construct was subcloned into pCDNA3.1-THAP to generate the THAP-mYFP-His6 construct and was transiently expressed in HEK293 cells. Cells were harvested into lysis buffer (10 mM Tris at pH 7.9, 0.1% Triton X-100, 150 mM NaCl, 2 mM EDTA, 5 μg/ml pepstatin, 1 mM PMSF, 1 mM NaF, and 1 mM Na_3_VO_4_) and lysates were serially diluted, then applied for single-molecule pull-down analysis. THAP-mYFP-His6 from lysate was immobilized on biotinylated anti-His (Qiagen), anti-THAP, anti-EGFP (Rockland Immunochemicals) or GBP1_6-Fc, or on biotinylated anti-HA as a negative control.

Anti-THAP and GBP1+6-Fc were labeled with EZ-Link Hydrazide-PEG_4_-Biotins (Thermo Scientific) according to the manufacturer’s manual. The concentration of the biotinylated proteins and biotin incorporation were determined by a Nanodrop spectrophotometer (Thermo Scientific).

EGFR was amplified by PCR from a plasmid containing full length EGFR coding sequence (kindly provided by Dr. Colleen Sweeney, UCDMC) and inserted into the mYFP construct. The EGFR-mYFP construct was transiently expressed in HEK293 and CHO cells with different DNA amounts as indicated for 12 hours in serum-free medium, and then treated with or without 10 mM EGF ligand immediately before harvesting cells. Cells were harvested into lysis buffer (10 mM Tris pH 7.9, 0.1% n-dodecyl-D-maltoside (DDM), 150 mM NaCl, 2 mM EDTA, 5 μg/ml pepstatin, 1 mM PMSF, 1 mM NaF, 1 mM Na_3_VO_4_) and lysates were applied for single-molecule pull-down analysis. Proteins were immobilized on biotinylated GBP1+6-Fc or anti-GFP antibody.

## Results

### Tandem GCN4 epitope and single-chain antibody fragment/Fc fusion

**Fig 2:**
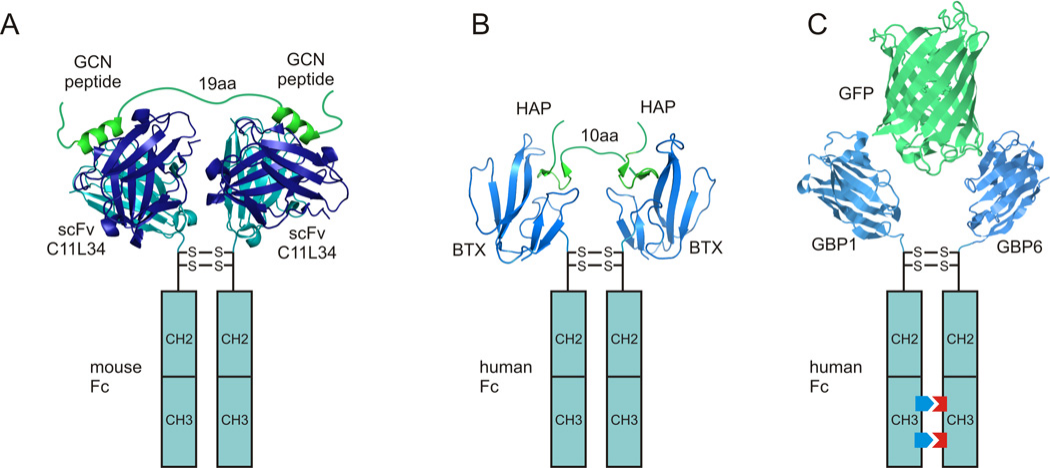
Strategy for designing high-affinity antibodies. (**A**) Schematic of the scFv C11L34-Fc fusion binding to a tandem GCN epitope. The recombinant antibody has identical Fc regions and is homodimeric. (**B**) THAP and anti-THAP. Bungarotoxin (BTX) replaces the variable region. (**C**) Recombinant bi-specific antibody to EGFP, created by different mutation of each Fc region to force heterodimeric binding. Each half of the dimer incorporates a nanobody (GBP1 or GBP2) targeting different regions of EGFP.

We first developed an antibody/epitope pair using a peptide antigen derived from the leucine zipper domain of the yeast transcription factor GCN4. Berger et al. (1999) [12] [13] raised antibodies to a 33-amino-acid GCN4 peptide and used directed, in vitro evolution to derive a single-chain antibody fragment (scFv; clone C11L34) with high affinity. To further increase avidity, we engineered a divalent antibody with the C11L34 scFv fused to a mouse Fc fragment (Fig 2A; two related constructions in Fig 2B and 2C are discussed below).

**Fig 3:**
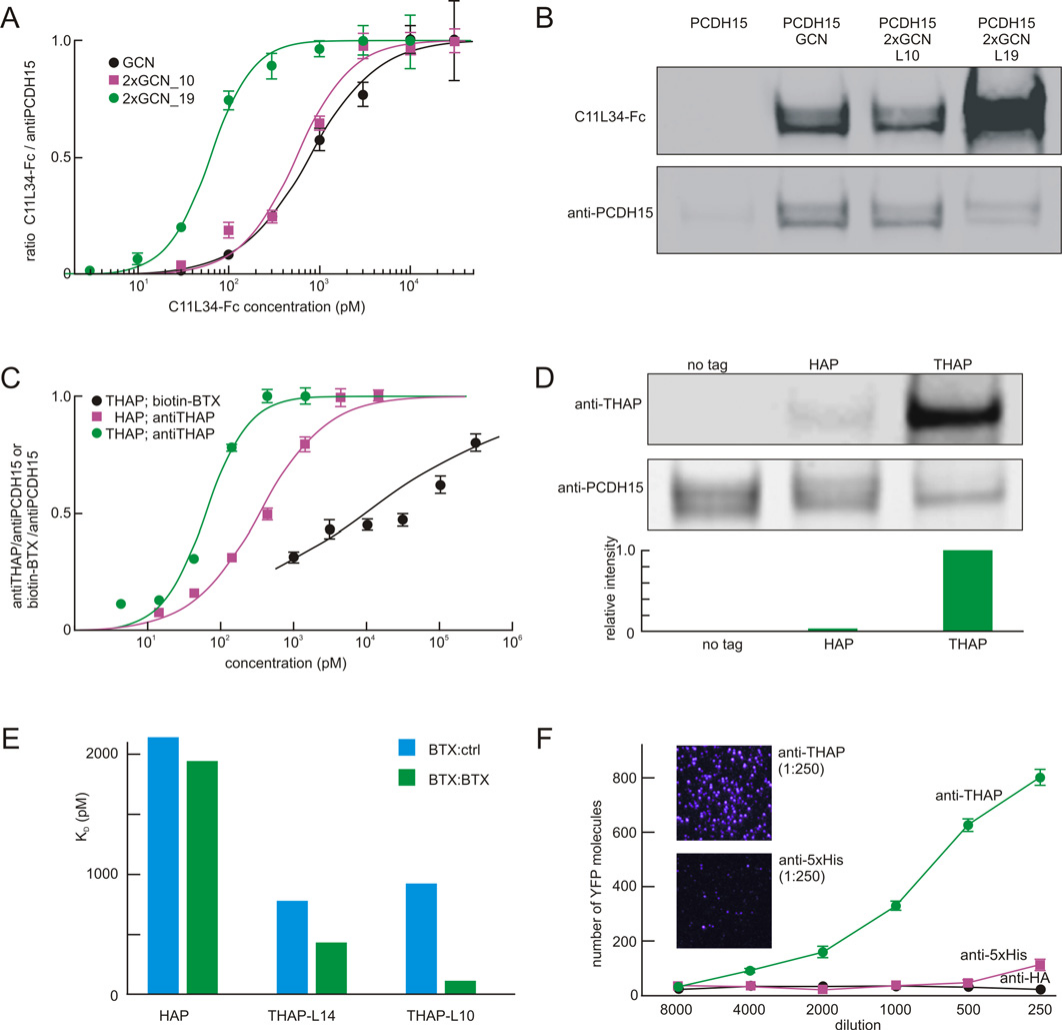
Performance of homodimeric antibodies with tandem epitopes. (**A**) Binding of recombinant C11L34 antibody to a single or tandem GCN epitope placed within the PCDH15 sequence and expressed in RPMI 2650 cells. Binding was normalized to the signal of an anti-PCDH15 antibody. The binding to the tandem epitope was about 15x better, if the linker was 19 amino acids (green line) but not if the linker was shorter (magenta). (**B**) Immunoblots of cell extracts showing much stronger binding to the tandem epitope with longer linker. Lysates from HEK cells expressing PCDH15 with different tags were run on two identical gels; one was probed with the divalent C11L34 anti-GCN at a fixed concentration and one with anti-PCDH15 as a loading control. Full sized immunoblots are in S3 Fig. (**C**) Binding of recombinant anti-THAP antibody or biotinylated BTX to a single or tandem HAP epitope placed within the PCDH15 sequence, normalized to the anti-PCDH15 signal. Anti-THAP avidity for the THAP tag was about 7X better than to the single HAP tag. (**D**) Immunoblots of cell extracts showing much stronger binding to the tandem epitope than to the single HAP. Protocol as in (B). Lower panel shows the binding to the tagged PCDH15 by anti-THAP relative to anti-PCDH15. Full sized immunoblots are in S4 Fig. (**E**) Biacore surface plasmon resonance analysis of binding between the recombinant antibodies to HAP or THAP peptides. One antibody (anti-THAP; shown as BTX:BTX) contained symmetric BTX binding domains, while another (BTX:con) was asymmetric with one arm containing BTX and the other an irrelevant control binding domain. The THAP peptide showed higher affinity, but it was substantially higher only for the anti-THAP antibody, and only when the linker length was 10 rather than 14 amino acids. Representative traces are in S1 Fig. Measured rate constants and dissociation constants are in S1 Table. (**F**) Single-molecule pull-down assay. Cell lysates containing monomeric YFP tagged with THAP and 6xHis were applied at different dilutions to slides with immobilized anti-His or anti-THAP antibodies, or anti-HA as a negative control. Insets show molecules pulled down. Full immunoblots for panels B and D are shown in S3 and S4 Figs.

We then made a tandem epitope with two copies of a 12-amino-acid fragment of the original peptide (AHLENEVARLKK; termed GCN), separated by either a 10- or 19-amino-acid linker. We tagged a membrane protein of interest, mouse protocadherin-15 (PCDH15), by inserting either a single or tandem GCN epitope into its extracellular domain. The tagged PCDH15 was expressed in RPMI 2650 cells, and fixed or unfixed cells were labeled with a conventional antibody to PCDH15 at a fixed concentration and with the C11L34-Fc fusion antibody at variable concentration. We then plotted the ratio of C11L34-Fc antibody fluorescence to anti-PCDH15 fluorescence to calculate avidity. The K_d_ of C11L34-Fc for a single GCN epitope was about 900 pM (Fig 3A). The avidity for a tandem GCN epitope was about the same, when the linker was 10 amino acids. With a longer 19-aa linker, however, the effective K_d_ was ~60 pM, so the shorter linker apparently prevented the second C11L34 scFv from reaching the second GCN.

We also tested the same constructs with immunoblots (Fig 3B; S3 Fig). As with immunocytochemistry, the tandem epitope with 19-aa linker markedly increased binding compared to the single epitope or the tandem epitope with shorter linker. In general, the avidity was increased by more than an order of magnitude by dimerizing the C11L34 EBD and by constructing a tandem epitope with the appropriate linker.

Dimerizing EBDs with Fc’s has several advantages in addition to avidity: Fc fusion proteins can be recombinantly expressed, with good protein yield and solubility. Also, the Fc fragment is a convenient handle, which enables signal amplification (through secondary antibodies), and chemical conjugation (to fluorophores, magnetic beads, gold particles, surface immobilization, etc.) [14]. Finally, by using Fc fragments from different IgG classes and different species, a variety of reagents with the same EBDs can be generated, enabling great flexibility in imaging experiments where multiple components are studied.

### THAP/anti-THAP system

Additional performance can be gained by using naturally-occurring peptide/ligand pairs as a starting point for a tandem epitope/antibody system, because they can be smaller and more stable. One such pair is α-bungarotoxin (BTX) and the peptide to which it binds. Bungarotoxin, isolated from the snake *Bungarus multicinctus*, is a 74-amino-acid protein that binds the acetylcholine receptor (AChR) of skeletal muscle with high affinity and specificity [15]. The BTX binding site on the AChR is an antiparallel β-hairpin structure of about 13 amino acids. Although BTX has been used with the epitope derived from its binding site in the AChR, short peptide sequences with higher affinity for BTX have been isolated [16] [17]. One of these peptides, a 13-amino acid peptide (WRYYESSLEPYPD) called high affinity peptide (HAP), has the highest affinity of those identified—about 2 nM. It forms an antiparallel β-hairpin structure like the native ligand [16]. HAP, in combination with fluorophore-conjugated BTX, has been used to label a variety of proteins in both fixed and live cells [8, 18].

Although the HAP/BTX is a useful system for cellular imaging, its affinity and specificity are not ideal. The apparent affinity of the HAP/BTX interaction in real imaging experiments is 14–60 nM rather than the 2 nM of biochemical experiments [8, 18], with a significant background. We suspect the loss of affinity and specificity in imaging experiments is due in part to the conjugation of fluorophores to BTX through its primary amine groups (lysine side chains and N terminus), which may inhibit binding. In addition, there is no further signal amplification with labeled toxin, as there can be with secondary antibodies. The lack of signal amplification makes it difficult to use the HAP/BTX system in demanding situations such as single molecule imaging, which require higher signal-to-noise ratio and specificity. The amplification issue can be addressed by conjugating BTX to biotin and using streptavidin as a secondary detection step. However, the reduced affinity/specificity issue remains.

We therefore made the HAP tag divalent by placing two HAPs in tandem (THAP) with a flexible linker of 10 or 14 amino acids. The linker length was based on the crystal structure of BTX/HAP [19], assuming that each amino acid can extend about 0.3 nm. We made the BTX divalent by fusing it with the Fc portion of human IgG2b, which naturally dimerizes through multiple disulfide bonds [14], to create a divalent antibody we term anti-THAP (Fig 2B).

We again used immunofluorescence to compare the avidity of HAP/BTX and THAP/anti-THAP. We inserted HAP and THAP sequences into the extracellular domain of PCDH15 and transiently expressed the tagged proteins in RPMI 2650 cells. Immunofluorescence of the THAP reagent was compared to that of a fixed concentration of anti-PCDH15 (see METHODS). As shown in Fig 3C, biotinylated BTX binding to the tandem THAP had a dissociation constant of ~10 nM in these conditions. Anti-THAP binding to PCDH15 with a single HAP had a dissociation constant of ~0.4 nM. As both are monomeric binding reactions, the better affinity of BTX when fused to Fc (anti-THAP) may result from a BTX binding surface unmodified by biotin. The effective affinity of the dimeric THAP/anti-THAP pair, however, is about 70pM, about 7-fold higher than the HAP/anti-THAP and 500-fold higher than the HAP/BTX. We stained the cells under live and fixed conditions, which give similar results.

We also tried the THAP/anti-THAP pair in immunoblots (Fig 3D; S4 Fig). The THAP tag in PCDH15 generated a signal that is about 30-fold that of HAP when probed with anti-THAP, highlighting the power of divalent interaction.

To directly measure the affinity between THAP and anti-THAP, we performed surface plasmon resonance analysis, using THAP peptides with different linkers (Fig 3E; S1 Fig; S1 Table). The interaction of anti-THAP with THAP incorporating a 14-aa linker (THAP_L14) has an effective dissociation constant of ~430 pM, and interaction with THAP_L10 has an effective dissociation constant of ~100 pM, similar to that obtained with the immunofluorescence assay and about 20 fold lower than the monovalent HAP/BTX interaction of ~2000 pM. With this tandem epitope as well, linker length is an important determinant of avidity.

Finally, we used a single molecule pull-down assay [11] to analyze THAP/anti-THAP interaction (Fig 3F). We compared THAP/anti-THAP with the commonly used 6XHis /anti-His system. Different antibodies with same concentration were immobilized on the surface of microscopy slides, and cell lysate containing a fusion protein with both THAP and 6XHis tags was serially diluted and applied to the slides for counting individual proteins. With the same diluted cell lysates, anti-THAP pulled down much more fusion protein than anti-His. Anti-THAP is about 16 times more effective than anti-His antibody in this assay.

### GBP1+6-Fc: a bi-specific antibody against GFP

The THAP/anti-THAP system requires engineering a THAP tag into a protein. Often a high-affinity antibody is needed for a native protein. We extended the bivalent concept to create a system in which two native epitopes close to each other in a protein are recognized by a bi-specific reagent with different EBPs.

Sera of certain species contain both conventional IgGs as well as unusual single chain antibodies (sdAb)—sometime termed nanobodies. These species include camels, dromedaries, llamas and alpacas, and so the sdAbs have also been termed camelid antibodies. SdAbs are small, have good solubility and have good thermal stability. The variable domains of sdAbs (VH; 12–15 kD) can be expressed recombinantly [20].

Fc fusion proteins can be made heteromeric by engineering the CH3 domain in the Fc segment[6]. We reasoned that if two nanobodies bind independently to two different parts of the protein, we could generate a heteromeric, bi-specific nanobody-Fc fusion protein. The bi-specific nanobody-Fc should have better specificity and avidity than conventional IgGs, because there would be two arms binding the same protein in different places, in the same way that the divalent anti-THAP binds to two HAP epitopes in tandem. Although bi-specific (“bi-paratopic”) nanobodies have been reported that have improved affinity when the two nanobodies are connected with a flexible linker[3], lack of a “handle” (for purification, chemical conjugation and signal amplification) limits their use in many antibody-based applications.

**Fig 4:**
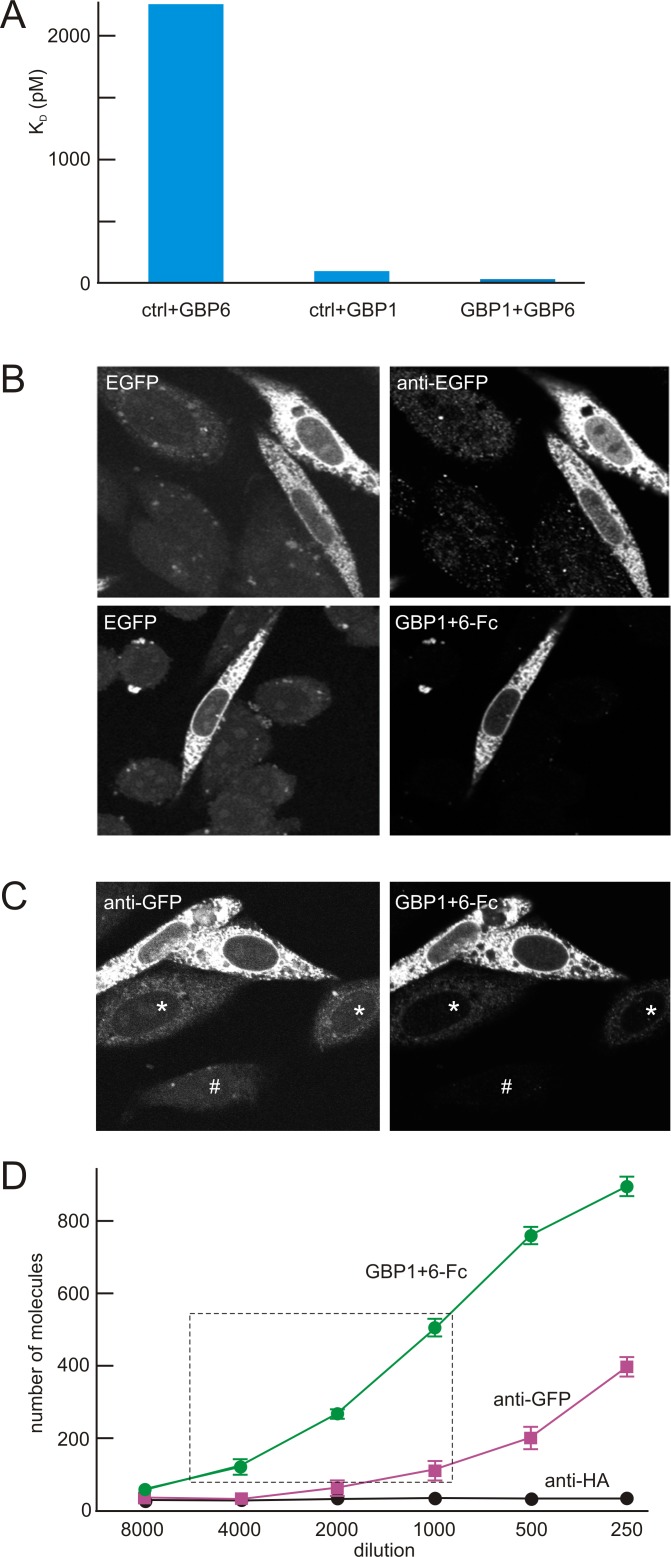
Binding of heterodimeric GBP antibody to EGFP. (**A**) Biacore surface plasmon resonance assay for effective affinity. Three test antibodies contained a GBP6-Fc fusion with an irrelevant Fc control arm (crtl+GBP6), a GBP1-Fc with an irrelevant Fc (crtl+GBP1), and a heterodimeric GBP1+GBP6 Fc fusion (GBP1+GBP6). Different concentrations of recombinant EGFP, ranging from 0 to 10 nM, were injected over immobilized Fc fusion proteins. Representative traces are in S2 Fig; rate constants in S1 Table. (**B**) CHO cells expressing EGFP assessed with EGFP fluorescence or anti-EGFP immunocytochemistry. CHO cells were transfected with EGFP fused to the transmembrane protein TMC1 as a carrier. Left panels show intrinsic EGFP fluorescence; right panels show label with either stock rabbit anti-GFP (top) or GBP1+6-Fc (bottom) with appropriate secondary antibodies. Imaging of the native EGFP fluorescence revealed bright EGFP-expressing cells, but also autofluorescence of non-transfected cells (top left). An anti-GFP antibody signal, adjusted for the same brightness, had less but not zero non-specific signal (top right). In comparison, the GBP1+6-Fc antibody, adjusted for the same brightness, had no detectable nonspecific label (bottom right). (**C**) Direct comparison of rabbit anti-GFP and GBP1+6-Fc. Cells expressing TMC1_EGFP were labeled with both rabbit anti-GFP and GBP1+6-Fc and with appropriate secondary antibodies of different colors, adjusted for equal brightness. Both labeled two strongly-expressing cells (upper part of field) and two weakly-expressing cells (middle, *), but the anti-GFP showed more nonspecific label—visible in the nuclei of the middle cells and in a non-expressing cell (bottom, #). (**D**) Single-molecule pull-down. Cell lysates containing tagged monomeric YFP were applied to slides coated with anti-HA (control), anti-GFP, or GBP1+6-Fc. The dashed box shows the optimal range for single-molecule pull-down analysis; GBP1+6-Fc is substantially better than anti-GFP in this range.

Several nanobodies, GBP1-7, against enhanced green fluorescent protein (EGFP) have been generated and characterized [21]. Of these, GBP1 and GBP6 bind to EGFP independently [22]. We generated a heterodimeric Fc fusion protein, GBP1+6-Fc, by separately fusing GBP1 and GBP6 to oppositely polarized Fc fragments that can only bind to each other and not homodimerically (Fig 2C). We used a Biacore-3000 analyzer to measure interaction between EGFP and GBP1-Fc, GBP6-Fc, and GBP1+6-Fc (Fig 4A; S2 Fig; S1 Table). GBP1+6-Fc binds to EGFP with an effective dissociation constant of 32 pM, about 3-fold less than that of GBP1-Fc and 18-fold less than reported for bacterially expressed GBP1 [21]. The difference in measured affinity between bacterially expressed GBP1 and GBP1-Fc is probably due to differences in their conjugation to the chip surface: the bacterially expressed GBP1 was conjugated using amine-based conjugation [21], which could interfere with its interaction with EGFP, whereas the GBP1-Fc was conjugated through the Fc portion (using an anti-human Fc antibody), which left the GBP1 intact.

We then evaluated the specificity of GBP1+6-Fc in immunostaining experiments. In fixed CHO cells expressing a membrane protein of the endoplasmic reticulum (TMC1) tagged with EGFP or mCherry, GBP1+6-Fc labeled the EGFP-, but not mCherry-tagged protein. Compared to a good commercially available anti-GFP antibody (Life Technologies A-11122), GBP1+6-Fc produced brighter signal with less background (Fig 4B). In addition, GBP1+6-Fc labeled cells with very low EGFP expression (Fig 4C). Because GBP1 increases the EGFP signal by several fold upon binding to the fluorescent protein [21], the signal-to-noise ratio of GBP1+6-Fc could be even higher.

We also compared GBP1+6-Fc with the anti-GFP antibody in single molecule pull-down experiments. Because GFP and variants such as YFP share structure and most sequence, GBP1+6-Fc should bind all of them. Indeed, GBP1+6-Fc is about 6 times more sensitive than anti-GFP antibody in pulling down the same number of YFP fusion proteins (Fig 4D).

To further explore the efficacy of GBP1+6-Fc, we used this antibody to detect the dynamics of epidermal growth factor receptor (EGFR) before and after ligand binding. EGF binds to the monomeric form of EGFR and induces EGFR dimerization for signaling transduction [23]. However, the existence of preformed dimeric EGFR in quiescent cells is controversial. Although ligand-independent dimerization was reported in cancer or transfected cells with large amounts of receptor [24] [25], protein over-expression might lead to the formation of aggregates [26] [27], and artificial formation of dimeric EGFR. We therefore transfected YFP-tagged EGFR at very low levels (50 ng/10^6^ cells, 10–40 times less than normal), which was close to the physiological condition in normal cells but fell below the detection limits of anti-GFP antibody. With GBP1+6-Fc, however, our single-molecule imaging method was still able to pull down EGFR, while the anti-GFP antibody could not (data not shown). Through a step-wise photobleaching-based analysis described previously [11], we showed that about 20% of EGFR was dimeric in the absence of ligand, indicating that dimerization is not due to receptor over-expression.

## Discussion

We have described a simple, practical approach to generate engineered antibodies with increased affinity and specificity. In the reagents described here, the two epitopes are covalently bound (as tandem tags or epitopes in different part of the same protein) and the two EBDs are covalently bound (through disulfide bonds on the Fc domains) to enhance binding and create very high affinity. Bi-specific antibodies are not new, yet they have generally been used to bind to two separate epitopes that are on different proteins or cells, in order to bring them together. Here, they produce high affinity for a single protein. The resulting antigen/antibody systems have greatly improved affinity, specificity and versatility.

Dimerizing EBDs with Fc’s has several advantages in addition to avidity: Fc fusion proteins can be recombinantly expressed, with good protein yield and solubility. Also, the Fc fragment is a convenient handle, which enables signal amplification (through secondary antibodies), and chemical conjugation (to fluorophores, magnetic beads, gold particles, surface immobilization, etc) [14]. Finally, by using Fc fragments from different IgG classes and different species, a variety of reagents with the same EBDs can be generated, enabling great flexibility in imaging experiments where multiple components are studied.

To our knowledge, the anti-THAP is by far the smallest (65 kD) anti-tag antibody with highest affinity (70–100 pM) to the corresponding peptide tag. Other commonly used anti-tag antibodies have lower affinity, such as the anti-HA antibody (clone 2–2.2.14, effective dissociation constant 4.5 nM), anti-Flag antibody (clone M2, 6.5 nM) [28], anti-myc antibody (clone 9E10, 80 nM) [29], or anti-His antibody (Qiagen 34440, 3.0–7.5 nM) [30]. It also has excellent stability: since BTX is resistant to boiling and strong acids, the stability of anti-THAP—a fusion of BTX and Fc—is determined by the stability of the IgG Fc portion.

Obviously this approach could be used to generate tandem epitopes for existing antibodies, such as anti-HA or anti-Flag; indeed, such epitopes are often expressed as multiples. To maximize affinity, the epitopes should be separated by a flexible linker, and linker length should be optimized. The anti-THAP reagent is smaller and more thermostable, however, and—as a recombinant protein—more reproducible.

The heteromeric, bi-specific Fc fusion protein with two nanobodies represents a simple and general approach to engineering high-affinity recombinant antibodies. Since nanobodies are small (12–15 kD), multiple nanobodies can bind to the same protein without interfering with each other. In the case of EGFP, a 27 kD globular protein, two nanobodies can bind to it simultaneously. Since many proteins are bigger and contain multiple structural domains, and since nanobodies generally have higher affinity and specificity than conventional antibodies, engineered antibodies containing multiple nanobodies (targeting different parts of the same protein) could easily be generated. These engineered antibodies should have much better affinity and specificity.

## Supporting Information

S1 Fig Surface plasmon resonance traces for BTX/HAP antibody/epitope pairs. Representative data for Fig 3E. CM5 sensor chips were coupled with goat anti-human IgG. Then human Fc fusion proteins containing either one BTX (BTX+control) or two BTX (BTX+BTX) were bound to the chip surface through the human Fc/goat anti-human IgG interaction. To measure affinity, solutions containing concentrations of each of the peptides (HAP, THAP_10, THAP_14), ranging from 0 to 10 nM, were injected over immobilized Fc fusion proteins.
(PDF)

S2 Fig Surface plasmon resonance traces for GBP/GFP antibody/target pairs. Representative data for Fig 4A. CM5 sensor chips were coupled with goat anti-human IgG. Then human Fc fusion proteins containing either GBP1, GBP6, or both were bound to the chip surface through the human Fc/goat anti-human IgG interaction. To measure affinity, solutions containing concentrations of recombinant EGFP, ranging from 0 to 10 nM, were injected over immobilized Fc fusion proteins.
(PDF)

S3 Fig Full gel of Fig 3B. Immunoblot of cell extracts showing much stronger binding to the tandem epitope with longer linker. Lysates from HEK cells expressing PCDH15 with different tags were run on two identical gels; one was probed with the divalent C11L34 anti-GCN at a fixed concentration and one with anti-PCDH15 as a loading control. Boxes indicate regions shown in Fig 3B.
(PDF)

S4 Fig Full gel of Fig 3D. Immunoblot of cell extracts showing much stronger binding to the tandem HAP (THAP) epitope than to the single HAP. Lysates from HEK cells expressing PCDH15 with different tags were run on two identical gels; one was probed with anti-THAP at a fixed concentration and one with anti-PCDH15 as a loading control. Boxes indicate regions shown in Fig 3D.
(PDF)

S1 Table On rates, off rates and avidities for antibody/ epitope pairs based on surface plasmon resonance. SPR traces were analyzed using BIAevaluation 4.1 software, which calculated rates and K_d_ of each interaction pair.
(PDF)
