# Supplementary material for: Three Recombinant Engineered Antibodies against Recombinant Tags with High Affinity and Specificity
Source: PLoS One. 2016 Mar 4;11(3):e0150125. doi: 10.1371/journal.pone.0150125 (PMC4778845; doi:10.1371/journal.pone.0150125)
Supplement: S1 Fig — Representative data for Fig 3E. CM5 sensor chips were coupled with goat anti-human IgG. Then human Fc fusion proteins containing either one BTX (BTX+control) or two BTX (BTX+BTX) were bound to the chip surface through the human Fc/goat anti-human IgG interaction. To measure affinity, solutions containing concentrations of each of the peptides (HAP, THAP_10, THAP_14), ranging from 0 to 10 nM, were injected over immobilized Fc fusion proteins. (PDF) [file pone.0150125.s001.pdf]

BTX+control/ HAP

BTX+control/ THAP-L14

BTX+control/ THAP-L10

BTX+BTX/ HAP

BTX+BTX/ THAP-L14

BTX+BTX/ THAP-L10

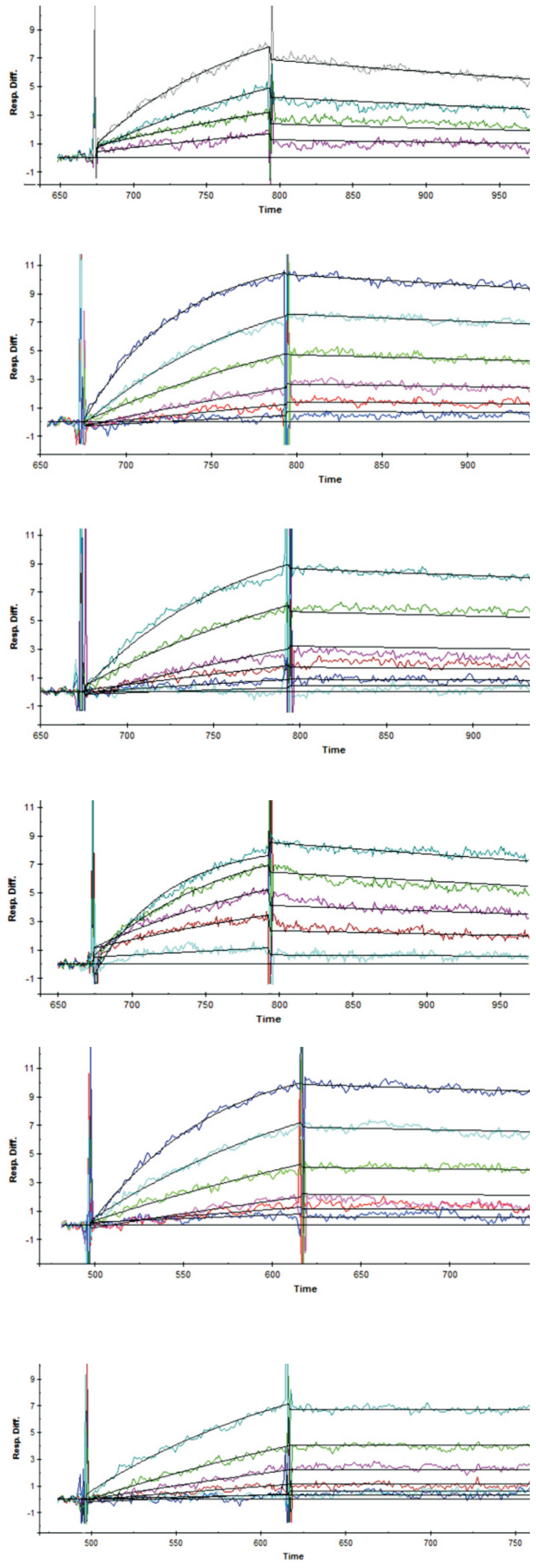

S1 Figure. Surface plasmon resonance traces for BTX/HAP antibody/epitope pairs. Representative data for Fig. 3E. CM5 sensor chips were coupled with goat anti-human IgG. Then human Fc fusion proteins containing either one BTX (BTX+control) or two BTX (BTX+BTX) were bound to the chip surface through the human Fc/goat anti-human IgG interaction. To measure affinity, solutions containing concentrations of each of the peptides (HAP, THAP_10, THAP_14), ranging from 0 to 10 nM, were injected over immobilized Fc fusion proteins.
