# Supplementary material for: Three Recombinant Engineered Antibodies against Recombinant Tags with High Affinity and Specificity
Source: PLoS One. 2016 Mar 4;11(3):e0150125. doi: 10.1371/journal.pone.0150125 (PMC4778845; doi:10.1371/journal.pone.0150125)
Supplement: S2 Fig — Representative data for Fig 4A. CM5 sensor chips were coupled with goat anti-human IgG. Then human Fc fusion proteins containing either GBP1, GBP6, or both were bound to the chip surface through the human Fc/goat anti-human IgG interaction. To measure affinity, solutions containing concentrations of recombinant EGFP, ranging from 0 to 10 nM, were injected over immobilized Fc fusion proteins. (PDF) [file pone.0150125.s002.pdf]

GBP1+control/ GFP

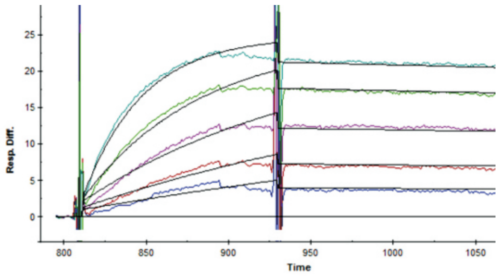

GBP6+control/ GFP

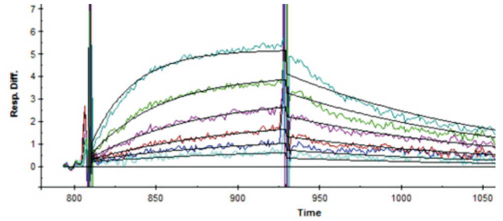

GBP1+GBP6/ GFP

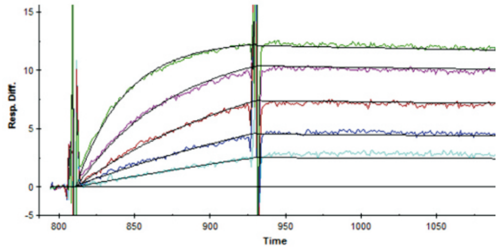

S2 Figure . Surface plasmon resonance traces for GBP /GFP antibody /target pairs. Representative data for Fig. 4A. CM5 sensor chips were coupled with goat anti-human IgG. Then human Fc fusion proteins containing either GBP1, GBP6, or both were bound to the chip surface through the human Fc/goat anti-human IgG interaction. To measure affinity, solutions containing concentrations of recombinant EGFP, ranging from 0 to 10 nM, were injected over immobilized Fc fusion proteins.
