# Supplementary material for: Three Recombinant Engineered Antibodies against Recombinant Tags with High Affinity and Specificity
Source: PLoS One. 2016 Mar 4;11(3):e0150125. doi: 10.1371/journal.pone.0150125 (PMC4778845; doi:10.1371/journal.pone.0150125)
Supplement: S3 Fig — Immunoblot of cell extracts showing much stronger binding to the tandem epitope with longer linker. Lysates from HEK cells expressing PCDH15 with different tags were run on two identical gels; one was probed with the divalent C11L34 anti-GCN at a fixed concentration and one with anti-PCDH15 as a loading control. Boxes indicate regions shown in Fig 3B. (PDF) [file pone.0150125.s003.pdf]

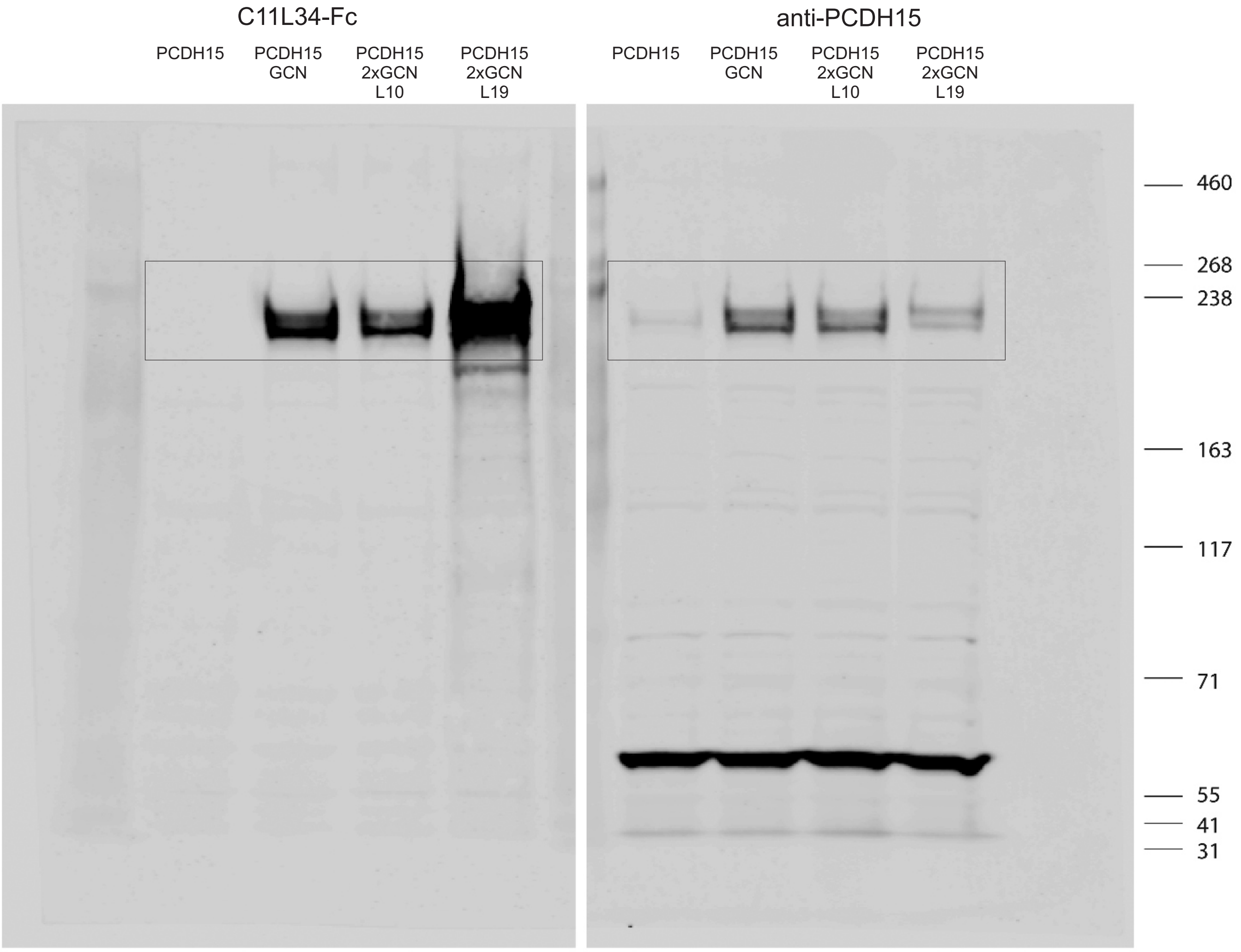

S3 Fig. Full gel of Fig 3B. Immunoblot of cell extracts showing much stronger binding to the tandem epitope with longer linker. Lysates from HEK cells expressing PCDH15 with different tags were run on two identical gels, one was probed with the divalent C11L34 anti GCN at a fixed concentration and one with anti PCDH 15 as a loading control. Boxes indicate regions shown in Fig 3B.
