# Supplementary material for: Three Recombinant Engineered Antibodies against Recombinant Tags with High Affinity and Specificity
Source: PLoS One. 2016 Mar 4;11(3):e0150125. doi: 10.1371/journal.pone.0150125 (PMC4778845; doi:10.1371/journal.pone.0150125)
Supplement: S4 Fig — Immunoblot of cell extracts showing much stronger binding to the tandem HAP (THAP) epitope than to the single HAP. Lysates from HEK cells expressing PCDH15 with different tags were run on two identical gels; one was probed with anti-THAP at a fixed concentration and one with anti-PCDH15 as a loading control. Boxes indicate regions shown in Fig 3D. (PDF) [file pone.0150125.s004.pdf]

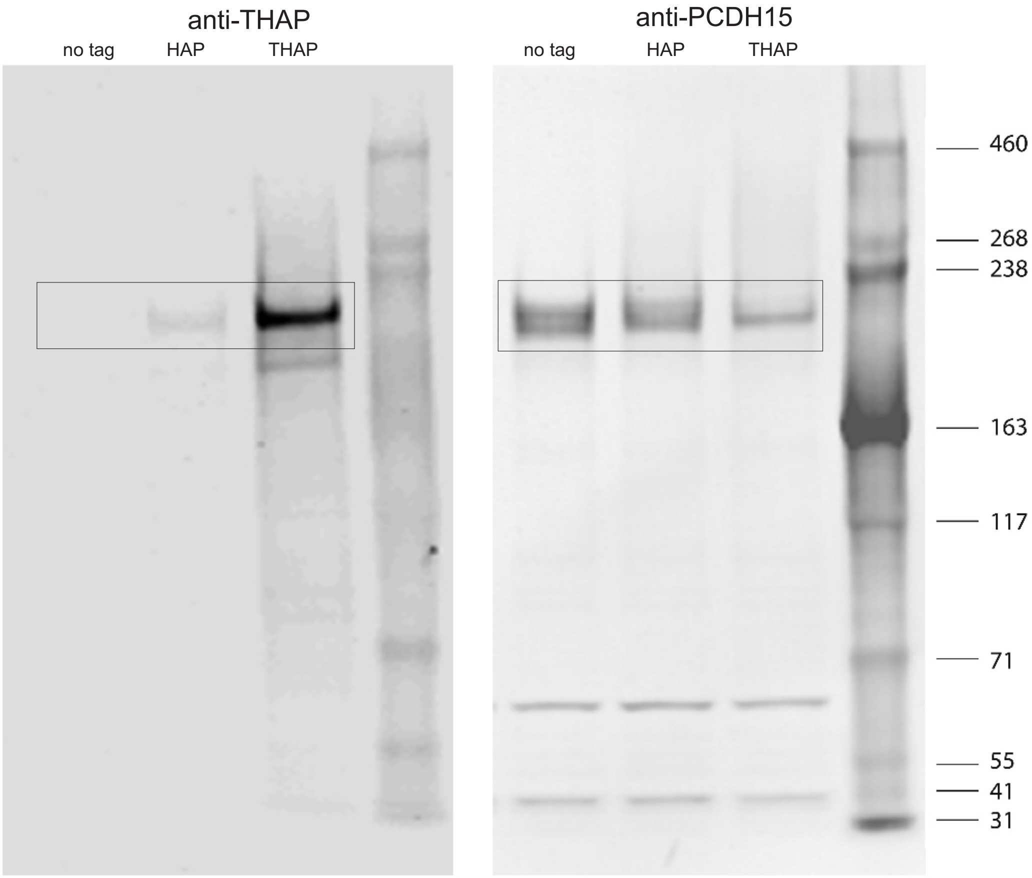

S4 Fig. Full gel of Fig 3D. Immunoblot of cell extracts showing much stronger binding to the tandem HAP (THAP) epitope than to the single HAP. Lysates from HEK cells expressing PCDH15 with different tags were run on two identical gels ; one was probed with anti-THAP at a fixed concentration and one with anti-PCDH15 as a loading control . Boxes indicate regions shown in Fig 3D.
