# Supplementary material for: Three Recombinant Engineered Antibodies against Recombinant Tags with High Affinity and Specificity
Source: PLoS One. 2016 Mar 4;11(3):e0150125. doi: 10.1371/journal.pone.0150125 (PMC4778845; doi:10.1371/journal.pone.0150125)
Supplement: S1 Table — SPR traces were analyzed using BIAevaluation 4.1 software, which calculated rates and Kd of each interaction pair. (PDF) [file pone.0150125.s005.pdf]

Pair	on rate ($M^{-1}s^{-1}$)	off rate (s^{-1})	K_d (M)	Rmax (RUx1000)
BTX+control/ HAP	4.0E+05	8.5E-04	2.1E-09	12
BTX+control/ THAP-L14	8.8E+05	6.8E-04	7.8E-10	12
BTX+control/ THAP-L10	6.5E+05	6.0E-04	9.2E-10	12
BTX+BTX/ HAP	4.9E+05	9.5E-04	1.9E-09	10
BTX+BTX/ THAP-L14	9.4E+05	4.1E-04	4.3E-10	12
BTX+BTX/ THAP-L10	3.5E+05	4.0E-05	1.1E-10	12
GPB1+control/ GFP	2.7E+06	2.8E-04	1.0E-10	22
GPB6+control/ GFP	3.2E+06	7.3E-03	2.3E-09	5
GPB1+GBP6/ GFP	6.1E+06	2.0E-04	3.3E-11	13

S1 Table. On rates, off rates and avidities for antibody/epitope pairs based on surface plasmon resonance. SPR traces were analyzed using BIAevaluation 4.1 software, which calculated rates and K_d for each interaction pair.
